# Knowledge, attitudes, and behaviors of parents towards varicella and its vaccination

**DOI:** 10.1186/s12879-017-2247-6

**Published:** 2017-02-27

**Authors:** Luigi Vezzosi, Gabriella Santagati, Italo F. Angelillo

**Affiliations:** Department of Experimental Medicine, Second University of Naples, Via Luciano Armanni, 5, 80138 Naples, Italy

**Keywords:** Attitudes, Behavior, Italy, Knowledge, Parents, Vaccination, Varicella

## Abstract

**Background:**

The aims of this cross-sectional survey were to examine the knowledge, the attitudes, and the behavior regarding the varicella infection and its vaccination and to get insight into their determinants among parents of children in Italy.

**Methods:**

From May to June 2015 in the geographic area of Naples (Italy) a random sample of 675 parents of children aged 4-7 years received a self-administered anonymous questionnaire about socio-demographic characteristics, knowledge, attitudes, and behaviors towards varicella and its vaccination.

**Results:**

A total of 414 parents responded to the questionnaire, for a response rate of 61.3%. A history of varicella was reported in 163 children (39.6%). Only 26.6% parents knew that the vaccine was available and the number of doses and this knowledge was significantly higher in those who had a university degree, in those who had received information on the vaccination from a health care provider, and in those who had vaccinated their child. The perceived utility towards vaccination had a mean value of 5.7. The positive attitude towards the utility of the vaccination was higher in parents with a level of education not higher than middle school, in those who had vaccinated their child, in those who considered the varicella a dangerous disease, and in those who had received information from a health care provider. More than one-third had vaccinated their child. Immunization was more frequent in parents who had knowledge about the vaccination, who beliefs that the immunization was useful, who believed that the disease was not dangerous, and who had not a history of varicella among their children.

**Conclusions:**

Educational programs are needed among parents as support to improve knowledge about vaccination and immunization coverage.

**Electronic supplementary material:**

The online version of this article (doi:10.1186/s12879-017-2247-6) contains supplementary material, which is available to authorized users.

## Background

It is well known that varicella is one of the most common and highly contagious viral diseases in childhood and it can be fatal especially in neonates due to its serious complications [1]. The adoption of childhood prophylactic vaccine against varicella-zoster virus has been shown to be safe and efficacious in reducing the incidence and morbidity associated [2].

In Italy the vaccine is licensed since 1994 [3] and the routine immunization has been introduced in the National Vaccination Plan 2012–2014 beginning with the 2014 birth cohort with the achievement and maintenance of vaccine coverage ≥95% for one dose within 2 years of age, and two doses in children aged 5–6 years of age [4]. However, the immunization is provided free of charge only in few geographic areas mainly because the National Health Service has been decentralized from the 2001 and the 20 regions are responsible for delivering public health and health-care services and, therefore, they may adopt different policies. In 2014, Campania Region had adopted a free of charge universal varicella vaccination programme targeting children and susceptible adolescents, according to the National Vaccination Plan [5].

However, the infant and childhood vaccination coverage unfortunately appear to be inadequate. Parents have an important and critical role in order to protect their children from acquiring and transmitting varicella by improving the coverage since their lack of knowledge and lower perception about the benefit of the vaccination could influence their choice for not vaccinating their son or daughter. Therefore, it is important to discover the perceptions linked to vaccination practices in order to improve parental awareness and coverage.

Different studies have shown that children’s parents could underestimate the incidence and severity of the varicella infection and that increasing awareness and knowledge about the disease and the vaccination are a key-factor to improve vaccine coverage [6–11]. So far, to the best that we could ascertain, little is known about this topic among parents of children in Italy. Therefore, the present investigation examined the level of knowledge of varicella infection and its vaccination, and the attitudes and behavior regarding the vaccination and to get insight into their determinants among parents of children in Italy.

## Methods

### Setting and participants

From May to June 2015, a cross-sectional survey was conducted in the city of Naples (Italy). Four kindergartens and primary public schools were randomly selected.

The head teacher of the selected kindergartens and schools was contacted by the research team and solicited for participation. After the approval, in each kindergarten and school, 8 classes were selected randomly and the sample was of 675 parents of children from 4 years of age until reaching 7 years of age. The minimum sample size required for the study was determined based on the assumptions of a prevalence of positive attitude regarding varicella immunization among parents of 50%, a 95% confidence interval, and a desired degree of accuracy of 0.05. To compensate the non-response rate, 40% of the determined sample was added up on the calculated sample size and the final sample size was 640.

### Procedure

The children selected received an envelope randomly addressed to either the mother or the father. The envelope included a letter with information on the study objectives, a two-page anonymous and confidential self-administered questionnaire, and a self-addressed envelope for returning the questionnaire to the research team. In the letter and at the beginning of the questionnaire, participants were assured of their privacy, that the survey was anonymous, and that the questionnaire responses were not linked with the participants’ identification. The letter also indicated that they received the questionnaire because their child was randomly selected in the kindergarten or in the school and they were given instructions to return the completed questionnaire to the kindergarten or the school within three days. If the questionnaire was not returned in the prescribed time, the research team made a reminder phone calls to the head teacher. No incentives were offered for completion of the survey. Respondents were never contacted directly by the research team.

### Questionnaire

A self-administered structured questionnaire was developed and pilot-tested among a convenience sample of 40 parents, who were interviewed to gain feedback on the overall acceptability of the questionnaire in terms of length, clarity, and question formats. The internal reliability was estimated through Cronbach’s α [12]. The questionnaire including 29 questions was structured in five sections [Additional file 1]. The first section involved questions regarding socio-demographic variables of the respondent (gender, age, marital status, degree of education, occupation, number of children) and of the selected child (age, birth order). The questions in the second section were about the knowledge about varicella, transmission route of the infection, and vaccination. The third section investigated the attitudes towards varicella and the vaccination. In the fourth section participants were asked to report whether they had vaccinated for varicella the selected child or any other child of at least 2 years old. Participants whose selected child was not vaccinated were asked whether they would or would not vaccinate their selected child or the other children in the future and subsequently giving reasons why or why not to vaccinate. Potential barriers, comprised a lack of information, concerns about efficacy, side effects, disapproval by the physician, and time constraints. Lastly, the fifth part assessed the awareness of the information about varicella and vaccination and the source of their information. Each part consisted of questions on a 10-grade Likert type scale ranging from 1 (Not worried, Not dangerous, Not useful) to 10 (Much worried, Very dangerous, Very useful), multiple-choice, and open-ended.

The Ethical Committee of the Second University of Naples approved the survey instrument and study protocol.

### Statistical analysis

Initially, to explore the association between the dependent variable and the independent variables, one at a time, a univariate analysis was performed. Chi-square test was used for comparisons of categorical variables, Student’s *t*-test was applied for continuous variables. Subsequently, if the outcomes of interest were associated with each independent variable with a *p* ≤ 0.25 in the univariate analysis, the variable was included in the multivariable linear and logistic regression models. The outcome variables were the following: knowledge regarding the availability of the varicella vaccine and the number of doses required (Model 1); positive attitude towards the utility of varicella vaccination (Model 2); and selected child’s vaccination uptake against varicella (Model 3). Independent variables tested at the univariate analysis for the inclusion into the models were the following: birth order and age of the selected child, age, marital status, educational level, number of children, knowledge about the disease (etiology, route of transmission), knowledge about the varicella vaccination (availability, dosage), perceived danger of disease, perceived utility of the varicella vaccination, information on the disease from a health care provider, information on the vaccination from a health care provider, need of additional information about varicella vaccination, history of varicella among at least one child, and vaccination of the selected child. Results from the stepwise linear regression models were presented as β-coefficients and standard errors. The results of the logistic regression models were presented as odds ratios (ORs) with 95% confidence intervals (95% CIs). The *p* value for variables entering in the logistic regression models was 0.2 and the *p* value to remain in the model was 0.4. All reported *p* values were based on two-tailed tests and were considered statistically significant at *p* = 0.05 or less. All data were analyzed using Stata version 10.1 statistical software [13].

## Results

Internal consistency reliability assessed using Cronbach’s α was 0.62. Of the 675 questionnaire distributed, a total of 414 parents participated for a response rate of 61.3%. As shown in Table 1, more than two-thirds were female, the average age was 40.4 years, the majority was married, for 49% of the respondents the selected child was first-born, the median number of children was two, one-third was small employers and own account workers, and approximately half attained a university degree.

**Table 1:** Self-reported characteristics of the participants in the study

	N	%
Gender (414)		
Male	113	27.3
Female	301	72.7
Age (years) (408)	40.4 ± 6 (22–58)^a^	
18–30	25	6.1
31–35	57	14
36–40	112	27.4
41–45	137	33.6
> 45	77	18.9
Marital status (411)		
Married	334	81.3
Other	77	18.7
Education level (411)		
No formal education, elementary or middle school	68	16.5
High school	147	35.8
College degree or higher	196	47.7
Socioeconomic status (395)		
Low	164	41.5
Middle	131	33.2
High	100	25.3
Number of children (414)		
1	88	21.3
2	227	54.8
≥ 3	99	23.9
Number of children by age (years) (414)		
4	50	12.1
5	68	16.4
6	151	36.5
7	145	35
Birth order of the selected children (414)		
First	203	49
Second	149	36
Third	48	11.6
Fourth and more	14	3.4
Varicella vaccination according to the age (years) of the selected children (156)^b^		
4–5	45	28.8
6–7	111	71.2

In brackets there is the number of parents reporting the information

Number for each item may not add up to total number of study population due to missing value

^a^ Mean ± standard deviation (range)

^b^ Only for the selected children who had been vaccinated

The vast majority of the parents answered correctly the statement that varicella is an infectious disease (91.9%) and that the transmission is mainly person to person by airborne respiratory droplets, direct contact with vesicle fluid of chickenpox cases and of patients with herpes zoster (68.9%). More than three-quarter (82.6%) knew that a vaccine is currently available, however, only 34.2% of the sample know the number of doses. Overall, participants were poor knowledgeable since 26.6% answered correctly that the vaccine was available and the number of doses required. A multivariable logistic regression analysis was conducted to establish a model with more than one variable that can predict the knowledge about the varicella vaccination. The analysis suggests that parents with a level of education not higher than middle school (OR = 0.41; 95% CI 0.17–0.99) were less likely to be knowledgeable in comparison with those who had a university degree, whereas those who had received information on the vaccination from a health care provider (OR = 2.35; 95% CI 1.14–4.85) and who had vaccinated their child against varicella (OR = 4.94; 95% CI 2.84–8.58) were more knowledgeable (Model 1 in Table 2).

**Table 2:** Multivariate analysis indicating associations between several variables and the outcomes regarding vaccination against varicella

Variable	OR	SE	95% CI	*p*
Model 1: Knowledge regarding the availability of the vaccine and the number of doses required				
Log likelihood = -186.82, ${\chi}^2$ = 81.42 (8 df), *p* < 0.0001				
Vaccination of the selected child	4.94	1.39	2.84–8.58	<0.001
Information on the vaccination from a health care provider	2.35	0.87	1.14–4.85	0.02
Educational level				
College degree or higher	1^a^			
High school	0.59	0.17	0.33–1.06	0.077
No formal education/elementary school/middle school	0.41	0.18	0.17–0.99	0.047
Age	1.05	0.03	0.99–1.10	0.073
Number of children				
One	1^a^			
Three or more	0.59	0.20	0.30–1.16	0.128
Knowledge about disease (etiology and transmission)	1.49	0.40	0.88–2.52	0.133
History of varicella among at least one child	0.71	0.20	0.41–1.24	0.237
Variable	Coeff.	SE	*t*	*p*
Model 2: Positive attitude towards the utility of varicella vaccination				
*F*(6,380) = 59.20, *R* ^2^ = 0.48%, adjusted *R* ^2^ = 0.47%, $p$ < 0.0001				
Perceived danger of the disease	0.43	0.05	8.54	<0.001
Vaccination of the selected child	3.45	0.27	12.55	<0.001
Educational level				
College degree or higher	1^a^			
High school	0.49	0.27	1.80	0.073
No formal education/elementary/middle school	1.43	0.37	3.91	<0.001
Information on the vaccination from a health care provider	0.71	0.30	2.39	0.018
History of varicella among at least one child	−0.50	0.26	−1.91	0.057
Variable	OR	SE	95% CI	*p*
Model 3: Selected child’s vaccination uptake against varicella				
Log likelihood = -149.72, ${\chi}^2$ = 215.36 (8 df), *p* < 0.0001				
Perceived utility of varicella vaccination	1.66	0.10	1.48–1.86	<0.001
Knowledge about the varicella vaccination	4.70	1.59	2.42–9.12	<0.001
History of varicella among at least one child	0.40	0.12	0.23–0.72	0.002
Perceived danger of the disease	0.85	0.05	0.75–0.96	0.01
Marital status	0.54	0.19	0.27–1.07	0.078
Information on the vaccination from a health care provider	1.95	0.84	0.83–4.56	0.125
Age	0.97	0.02	0.92–1.01	0.180
Information on the disease from a health care provider	0.71	0.28	0.33–1.53	0.384

^a^Reference category

The results regarding attitudes and beliefs indicated that only 2.2% of the parents believed that varicella could cause serious health problems with an overall mean value of 3.9 on a 1 to 10 scale, whereas the overall perceived utility towards vaccination as a way to protect their child was low, with a mean value of 5.7. Multiple linear regression analysis showed that parents with a level of education not higher than middle school, those who reported that they had vaccinated their child against varicella, those who considered the varicella to be a dangerous disease, and those who had received information on the vaccination from a health care provider were more likely to have a positive attitude towards the utility of varicella vaccination (Model 2 in Table 2).

A history of varicella was reported in 163 of the selected children (39.6%). More than one-third of the respondents (38.4%) stated that they had vaccination their child and almost two-thirds of them (60.5%) were actually encouraged by their health care provider to vaccinate. The results of the multivariate logistic regression analysis examining the variables associated with selected children’s vaccination uptake against varicella showed that four variables were independently predictive of the vaccination. The strongest predictor was the personal knowledge about the varicella vaccination, respondents with this knowledge had an almost 5 times greater odds of vaccination than non-knowledgeable respondents (95% CI 2.42–9.12). Parents who had not a history of varicella among their children (OR = 0.4; 95% CI 0.23–0.72), those who believed that the disease was not dangerous (OR = 0.85; 95% CI 0.75–0.96), and those who had the personal beliefs about the utility of the vaccination (OR = 1.66; 95% CI 1.48–1.86) were more likely to have vaccinated their child against varicella (Model 3 in Table 2). Of the parents who had not vaccinated against varicella their child, only 20.8% stated that they would be willing to vaccinate their child and the main reasons for vaccine acceptance were that it was useful (39.2%) and that the disease can give complications (23.5%), whereas the most reported reasons for non-acceptance of the vaccine were that their child had already get the varicella (44.2%), that the varicella was not dangerous (39.2%), and that the health care provider did not recommend the vaccination (23.6%).

Almost all participants, reported that they had ever heard of the varicella (99.3%) before study participation, and the most reported sources for advice were family or friends (80.5%), 74.2% a health care provider, and 17.5% through TV, radio, magazine or newspaper articles. For those who answered that they had heard of the vaccine (92%), 83.3% indicated a health care provider, 52.3% family or friends, and 13.1% TV, radio, magazine or newspaper articles. One-third of the parents surveyed (35.2%), whether they would be willing to vaccinate their child or not, desired more information regarding varicella vaccination.

## Discussion

The results of the present study have improved the understanding of the parental knowledge, attitudes, and practices toward varicella infection and vaccination for children in Italy.

Regarding knowledge related to varicella and its vaccine, sampled parents answered correctly that it is an infectious disease and about the way of transmission, but knowledge on the vaccine was seriously poor since only one fourth knew that one was available and correctly indicated the number of doses. This finding is of concern because the government has included varicella in its immunization program [4, 14, 15]. These results are in accordance to similar studies conducted in USA [16] and Canada [17], with respectively 73.3 and 68% who were aware about the availability of the vaccine. Moreover, high knowledge was found in Germany [18], where the 95, 94.2, and 95.5% knew the existence of the vaccine. Lastly in UK [19], only 26% of parents had heard about the availability of the vaccination and in the Hawaii [20] 32% said they had no knowledge about the vaccine. The finding that an adequate level of knowledge about the varicella vaccination was a significant predicting factor for vaccination acceptance supports the importance of the knowledge. This indicates that an educational campaign should also cover beliefs and behaviors associated with the acceptance of vaccination.

An important result from this study is that, despite the vaccine in the selected sample was not provided free of charge, 38.4% of the respondents stated that they had vaccinated against varicella their child. Similar results have been observed in Israel with an immunization rate of 35.2% [21] and 34.1% [22], whereas higher values have been found in Taiwan with 69% [23], in Greece with 87.5% [24] and 61.1% [25], and in USA with 68% [26]. Whereas, lower values have been found in India (27.1%) [27], in Canada (21–28%) [28], and in Poland (4.2%) [29]. The low value regarding the vaccination in the present study is a possible explanation of the low overall perceived utility towards varicella vaccination as a way to protect their child, with a mean value of 5.7 in a 10-point scale, and of only 20.8% stated that they would be willing to vaccinate their child. About the perceived utility towards varicella vaccination, a higher value was found in Turkey with a mean value of 3.68 in a 5-point scale [30] and in USA with 4.4 in a 6-point scale [31]. In the already mentioned survey conducted in Germany, 83–94% of parents who vaccinated their children believed that varicella vaccine was useful, while only 20–30% of those who did not vaccinate their children thought that the immunization was useful [18].

The findings from the multivariable analysis showed several interesting associations. The contribution of the socio-demographic variables to the outcomes of interest indicated that only respondent’s educational level remained in the multivariate analyses predicting two outcomes of interest. Among the respondents with a university degree, the level of knowledge regarding the availability of varicella vaccine and the number of doses was significantly higher compared to those with a lower level of education. Previous studies showed that those with higher education were more likely to know the vaccination [6, 7, 10, 22]. This may be explained by the fact that parents with higher education background may have more access to health information and thus achieve higher knowledge and this is also sustained by the positive association between knowledge and information acquired by health care providers. The importance of the poor knowledge regarding the vaccination was also highlighted by the fact that being well-informed was the most significant predictor of having vaccinated a child. Moreover, the parents’ educational level has also a significant impact on the attitude towards the utility of varicella vaccination with parents with a level of education not higher than middle school were more likely to have a positive attitude [32].

A remarkable finding was that the health care providers were one of the most frequently consulted information sources on the vaccination by the respondents, followed by mass media, and friends or family. As expected, information delivered from a health care provider is effective, because these respondents had a significantly greater level of knowledge about the vaccine compared with those who had received advice from other sources. The result that physician recommendation is a key predictor of knowledge about vaccination underline the fact that they are in an unique position in conveying and acquiring knowledge and in educating and recommending the vaccine. The findings further support that they are an important and trustworthy source of information for parents regarding childhood vaccinations. Thus, educating parents regarding the varicella and the vaccination to acquire information from health care providers is paramount in order to encourage such population to vaccinate their child. Other research has demonstrated the importance of the physician in determining parent’s knowledge of vaccination for themselves or their child and of preventive health behaviors [9, 11, 16–18, 20, 21, 32–43]. However, it should be noted that health care providers had no effect on the decision of the parents to vaccinate their child although those who had received information on the vaccination from this source were likely to have a positive attitude towards the utility of the vaccine. It is therefore essential that health care providers should be aware of their role in communicating with parents regarding varicella vaccine and they must take advantage of every encounter to inform parents in line with the advice of health authorities also because one of the most important reasons for declining vaccination was that they had not recommended it.

As in all similar epidemiological research, certain limitations in the study design should be considered. First, the data are cross-sectional and although we were able to identify associations between the outcomes of interest and certain study factors, caution should be taken when interpreting the findings owing to the nature of the study method employed that prevents us from making any statements regarding temporality and causal relationships. Second, due to the self-administered nature of the questionnaires, data on the vaccination status was not confirmed by other data sources and therefore recall and reporting bias may have been introduced. We are however confident that reliable estimates have been collected given that many participants indicated that their daughter/son did not receive the vaccine, so overestimation of uptake is unlikely, and that a child could only receive the vaccine with consent of an adult caregiver, so it is very likely that one parent accompanied them and thus knows the number of doses received. Third, potential for socially desirable answers may lead to reporting bias with a tendency to agree with statements when in doubt or to over-reporting acceptability of the vaccination. Because of the cross-sectional nature of the study, it is unclear whether parents with positive attitudes toward varicella vaccination will actually vaccinate their children in the future, although a self-administered anonymous questionnaire should have allowed parents to respond more accurately to the questions.

## Conclusions

To conclude, the current investigation suggests a substantial need for educational campaigns to disseminate knowledge about varicella among parents and repeated exposure to information from health care providers about varicella and vaccination may result in high rates of vaccination.

## Additional file

Additional file 1: Questionnaire. (DOCX 49 kb)

## Abbreviations

CI: Confidence interval
OR: Odds ratio
SE: Standard error
