# Supplementary material for: Knowledge, attitudes, and behaviors of parents towards varicella and its vaccination
Source: BMC Infect Dis. 2017 Feb 27;17:172. doi: 10.1186/s12879-017-2247-6 (PMC5327543; doi:10.1186/s12879-017-2247-6)
Supplement: Additional file 1: — Questionnaire. (DOCX 49 kb) [file 12879_2017_2247_MOESM1_ESM.docx]

1. **SOCIO-DEMOGRAPHIC CHARACTERISTICS OF THE RESPONDENT PARENT**

**I will ask you some questions to gather information about your socio-demographic characteristics**

**A1.** Respondent □ Mother □ Father

**A2.** How old were you on your last birthday? _________________

**A3.** What is your marital status? □ Married □ Separated\Divorced\Widowed □ Cohabitant □ Single (never married) □ Other _____

**A4.** What is your nationality □Italian □ Other, specify __________________

**A5.** What is your current occupation? ____________________________________________________________________________

**A6.** What is your highest educational level?

□ None □ Elementary school □ Middle school □ High school □ University degree

**A7.** What is the date of birth of your selected child? ____________

**A8.** What is the birth order of the selected child? □ First □ Second □ Third □ Other, specify ____________________

**A9.** If you have more than one child, what is the date of birth of your other children: First child____________; Second child_____________;

Third child ___________; Fourth child____________; Fifth child_____________; Other, specify ____________________

**If currently you are single, please go to section B.**

**A10.** How old were your husband\wife\partner on his\her last birthday? _________________

**A11.** What is the nationality of your husband\wife\partner? □Italian □ Other, specify _____________________________

**A12.** What is the current occupation of your husband\wife\partner? _____________________________________________________

**A13.** What is your husband’s\wife’s\partner’s highest educational level?

□ None □ Elementary school □ Middle school □ High school □ University degree

**B. KNOWLEDGE**

**I will ask you some questions to explore your knowledge related to varicella and its vaccination**

**B1.** In your opinion what kind of disease is varicella? □ chronic □autoimmune □metabolic □ infectious □ hereditary □ inflammatory

**B2.** How can varicella be transmitted? ____________________________________________________________________________

**B3.** Is there a vaccine available for varicella in Italy?

□ No **(Please go to section C.)** □ I do not know □ Yes, how many doses are recommended? □ 1 □ 2 □ 3 □ 4 □ I do not know

**B4.** At what age the varicella vaccine should be given? _____________________ □ I do not know

**C. ATTITUDES**

**I would like to know your attitudes towards varicella and its vaccination. Answer the following questions as truthfully as possible**

**(If your selected child has already got varicella, please go to C2.)**

**C1.** On a scale from 1 to 10, how much are you worried that your selected child might get varicella? (1 indicates that you are not worried, 10 indicates that you are very worried)

NOT WORRIED 1 2 3 4 5 6 7 8 9 10 VERY WORRIED

**C2.** On a scale from 1 to 10, how serious do you consider varicella? (1 indicates that you do not consider it serious at all, 10 if you consider it very serious disease)

NOT SERIOUS 1 2 3 4 5 6 7 8 9 10 VERY SERIOUS

**C3.** On a scale from 1 to 10, how useful do you consider varicella vaccination? (1 indicates that you do not consider it useful at all, 10 if you consider it very useful)

NOT USEFUL 1 2 3 4 5 6 7 8 9 10 VERY USEFUL

**D. BEHAVIOR**

**I am going to ask you some questions that are designed to gather information about your behavior**

**D1.** Has your selected child already contracted varicella? □ No □ Yes

**D2.** Did you vaccinate your selected child against varicella?

□ Yes **(more than one answer is allowed)**  □ No **(more than one answer is allowed)**

| **Reasons for having vaccinated your child against varicella** | **Reasons for not having vaccinated your child against varicella** |
| --- | --- |
| □ The vaccine protects against varicella | □ My child has already contracted varicella |
| □ The vaccines are useful in promoting my child’s health | □ I am afraid of vaccination’ consequences on health |
| □ My physician recommended this vaccine | □ My physician did not recommend this vaccine |
| □ Complications can occur from varicella | □ Varicella is not dangerous |
| □ I am in favor of the vaccination | □ I am not in favor of varicella vaccination |
| □ Vaccine is safe and effective | □ Vaccine is not safe and effective |
| □ The brothers and/or the sisters reported some complications due to varicella | □ The brothers and/or the sisters did not report complications due to varicella |
| □ Varicella can leave permanent skin scars | □ Difficult of access to immunization centers |
| □ Other (specify) _____________________________________ | □ Other (specify) __________________________________________ |

**(If you have not vaccinated your selected child yet, please go to D3.; if you have already vaccinated your selected child please go to D4.)**

**D3.** Would you vaccinate your selected child against the varicella?

□ Yes **(more than one answer is allowed)**  □ No **(more than one answer is allowed)**

| **Reasons why you would vaccinate your child against varicella** | **Reasons why you would not vaccinate your child against varicella** |
| --- | --- |
| □ The vaccine protects against varicella | □ My child has already contracted varicella |
| □ The vaccines are useful in promoting my child’s health | □ I am afraid of vaccination’ consequences on health |
| □ My physician recommends this vaccine | □ My physician do not recommend this vaccine |
| □ Complications can occur from varicella | □ Varicella is not dangerous |
| □ I am in favor of the vaccination | □ I am not in favor of varicella vaccination |
| □ Vaccine is safe and effective | □ Vaccine is not safe and effective |
| □ The brothers and/or the sisters reported some complications due to varicella | □ The brothers and/or the sisters did not report complications due to varicella |
| □ Varicella can leave permanent skin scars | □ Difficult of access to immunization centers |
| □ Other (specify) _____________________________________ | □ Other (specify) __________________________________________ |

**(If you do not have other children, please go to section E.)**

**D4.** Have your other children contracted varicella? First child □ No □ Yes; Second child □ No □ Yes; Third child □ No □ Yes;

Fourth child □ No □ Yes; Fifth child □ No □ Yes; Sixth child □ No □ Yes

**D5.** Have you vaccinated your other children against varicella? First child □ No □ Yes; Second child □ No □ Yes;

Third child □ No □ Yes; Fourth child □ No □ Yes; Fifth child □ No □ Yes; Sixth child □ No □ Yes

**(If you have vaccinated your other children, please go to section E.)**

**D6**. If you have not vaccinated your children against varicella, would you vaccinate them in the future?

□ Yes, for which reasons? _____________________________; □ No, for which reasons? ___________________________________;

**E. INFORMATION**

**I am going to ask you some questions to know the sources and need of information about varicella and its vaccination**

**E1.** Have you ever heard about varicella? □No **(If you have never heard about varicella, please go to E2.)**

□ Yes, from which sources did you receive information about varicella? **(more than one answer is allowed)**

□ Mass Media □ Physicians □ Friends/Family members □ Internet □ Other (specify_______________________)

**E2.** Have you ever heard about varicella immunization? □ No **(If you have never heard about varicella immunization, please go to E3.)**

□ Yes, from which sources did you receive information about varicella immunization? (**more than one answer is allowed**)

□ Mass Media □ Physicians □ Friends/Family members □ Internet □ Other (specify_______________________)

**E3.** Do you feel you need more information about varicella immunization? □ No □ Yes

**The questionnaire is finished, thank you for taking the time to respond and please return it to the kindergarten/school within seven days using the self-addressed envelope**
